# The Effect of Organizational Innovation Climate on Employee Innovative Behavior: The Role of Psychological Ownership and Task Interdependence

**DOI:** 10.3389/fpsyg.2022.856407

**Published:** 2022-06-20

**Authors:** Yutian You, Zhongfeng Hu, Jiawei Li, Youhan Wang, Mingli Xu

**Affiliations:** ^1^School of Politics and Public Administration, South China Normal University, Guangzhou, China; ^2^School of Finance and Trade, Guangdong Industry Polytechnic, Guangzhou, China; ^3^Research Center for Education and Social Integration in Guangdong-Hong Kong-Macao Greater Bay Area, Guangzhou, China; ^4^College of Education, Michigan State University, East Lansing, MI, United States; ^5^School of Innovation and Entrepreneurship, Guangdong Polytechnic Normal University, Guangzhou, China

**Keywords:** organizational innovation climate, employee innovative behavior, psychological ownership, task interdependence, moderated mediator

## Abstract

In today’s era of rapid development of science and technology, organizations are confronted with unprecedented opportunities and challenges. Employee innovative behavior has become the key element to promote organizational innovation and achieve sustainable competitive advantages. This study examines the relationship between organizational innovation climate and employee innovative behavior by focusing on the mediating role of psychological ownership and the moderating role of task interdependence. The survey data were collected from the matched samples of 326 employees and their direct supervisors from 13 enterprises in Guangdong Province, China. The results indicate that organizational innovation climate is positively related to employee innovative behavior and that psychological ownership plays a fully mediating role between them. For the moderating effects, task interdependence positively moderates the relationship between organizational innovation climate and employees’ psychological ownership. The results also reveal an indirect effect of organizational innovation climate on employee innovative behavior through psychological ownership. Theoretical and practical implications are also discussed.

## Introduction

Employee innovative behavior has long been regarded as the main element for enterprises to promote innovation development and achieve sustainable competitive advantages (Shanker et al., 2017). As global competition intensifies and the economic environment becomes progressively more uncertain, enterprises must continually innovate to maintain a competitive edge in the marketplace (Pieterse et al., 2010). Consequently, researchers and managers are increasingly concerned about cultivating employee innovation, and the climate dimensions, such as support and autonomy, were found to be effective predictors of innovation performance (Hunter et al., 2007). According to the organizational sensemaking theory, sensemaking formation can be considered as a cognitive and emotional process influenced by the social and contextual environment in which it occurs. Thus, it has been used to explain how individuals and groups give sense to their experiences in organizations (Cristofaro, 2021). Moreover, the individual sensemaking process must be translated into organizational behaviors to produce outcomes (Kaplan, 2008). Prior studies have investigated the relationship between organizational innovation climate and employee innovative behavior; however, the internal mechanism of employees’ psychological formation needs further investigation.

Studies have shown that supportive organizational climate nurtures employee innovation (Lee et al., 2011; Shanker et al., 2017; Awang et al., 2019), which is prominently connected with organizational philosophy, team support, leadership support, job flexibility, and resource provision (Madrid et al., 2014; Wallace et al., 2016; Luo et al., 2018). When employees feel creditable and respected in a positive innovation climate, they have a higher sense of belonging and responsibility to the organization, motivating them to be more engaged and innovative in their work. Such a relationship builds ground for further examination of topics related to organizational innovation. In line with Liu F. et al. (2019), we aim to confirm this relationship at the beginning, with the unique sample we had obtained. From there, we can consider more nuances and delve deeper into the more complicated mechanism underlying employee innovative behavior. Research on the determinants of employee innovative behavior has primarily focused on organizational structure/culture, organizational citizenship behavior, leadership style, and corporate incentives (Hammond et al., 2011; Hon and Chan, 2013; Phung et al., 2018; Liu Y. et al., 2019; Zhou et al., 2021) and less involved employee psychological perception and job characteristics under the influence of organizational climate. Accordingly, this study seeks to address this gap by incorporating specific aspects into our conceptual model: psychological ownership and task interdependence.

Although Vandewalle et al. (1995) theorized that psychological ownership had affected individual innovation, empirical evidence was rarely provided. Psychological ownership refers to the psychological state of “mine” or “ours” to the target (organization or task), which promotes one’s work attitude and behavior (Pierce et al., 2001). Employees who have higher levels of psychological ownership in the workplace are more likely to produce innovative outcomes (Yoon et al., 2020). Employees feel psychologically intertwined with their organizations and spontaneously internalize innovation as their own goals. The sense of psychological ownership often induces satisfaction with their job (Schulte et al., 2006; Avey et al., 2012) and is accompanied by a sense of responsibility, proactiveness, and organization-based self-esteem (Liu et al., 2012; Cheng et al., 2021) to seek creative solutions to problems and foster innovation behavior accordingly (Liu F. et al., 2019; Leyer et al., 2021). Meanwhile, prior studies have shown that the organizational innovation climate fulfills the employees’ needs for a sense of belonging (Hammond et al., 2011; Übius et al., 2013). From this perspective, organizational innovation climate may indirectly affect employee innovative behavior by employees’ psychological ownership, which is tested in this study.

Employee innovation is task-relevant and, thus, is inseparable from the interaction of organizational environment and job characteristics. Some scholars have investigated job-level moderators between climate and creative achievement, for instance, creativity required and the amount of discretion on job (Hunter et al., 2007), while the influence of task interdependence has been neglected. Task interdependence refers to the degree that employees in an organization need resources and information to complete tasks or the dependence on communication and cooperation among members of the organization (van der Vegt et al., 2001; Bachrach et al., 2006). As an essential characteristic in the work design (Campion et al., 1993; Lisak et al., 2022), task interdependence affects one’s internal motivation and innovation performance (Liu et al., 2011). Especially, the global economic shift has dramatically altered the nature of work, and one of the most conspicuous changes is that task interdependence of work in the organization has been greatly enhanced (Mathieu et al., 2017), which makes it necessary to consider the role of task interdependence as a moderator.

Furthermore, task interdependence may moderate the relationship between organizational innovation climate and employees’ psychological ownership. Strengthening work connection and interdependence among employees promotes positive emotional attitudes and stimulates the sense of responsibility (Ramamoorthy and Flood, 2004; Zhang and Min, 2021), which makes employees willing to put in more effort and work more actively for the organization’s goals accordingly. When the level of task interdependence is low, employees often fall into the isolated situation of “working alone” and gradually become psychologically alienated. Their perceived sense of belonging to, identification with, and possession toward their organization may decrease. Hence, this study extends the literature on the role of task interdependence in moderating the relationship between organizational innovation climate and psychological ownership. Above all, the mechanism underlying employee innovative behavior is investigated at the organizational, individual, and job levels in the study.

To address the aforementioned issues, we developed our conceptual model and four hypotheses. These hypotheses were tested on data collected from the highly innovative samples of 326 employees and their direct supervisors from 13 enterprises in Guangdong Province, China. The leader–member paired questionnaires were adopted to attenuate the common method bias. Evidence was then analyzed using Cronbach’s alpha, confirmatory factor analysis, and hierarchical regression analysis. The results showed that organizational innovation climate positively influenced employee innovative behavior and that psychological ownership played a fully mediating role between them. More importantly, task interdependence positively moderated the relationship between organizational innovation climate and employees’ psychological ownership. In addition to this, the indirect effect of organizational innovation climate on employee innovative behavior through psychological ownership was revealed.

This study aims to expand the existing literature as follows: First, in contrast with previous research (e.g., Jaiswal and Dhar, 2015; Shanker et al., 2017; Liu F. et al., 2019; Leyer et al., 2021), this study attempts to unfold the mechanisms through which organizational innovation climate affects employee innovative behavior, with leader–member paired questionnaires from a highly innovative sample in information transmission, information technology service, and financial industry to attenuate the common method bias. Second, drawing on the psychological ownership theory (Pierce et al., 2001, 2003; Van Dyne and Pierce, 2004), this study provides a new theoretical insights into the organizational climate–innovative behavior linkage by highlighting the positive role of psychological ownership in long-term organizational development from the perspective of employee innovative behavior, which is different from most of the literature on its role in employee organizational citizenship behavior (e.g., Jiang et al., 2019; Abbas et al., 2022). Third, by taking task interdependence as a moderating variable, this study responds to Hunter et al.’s (2007) call for paying more attention to the moderators in the organizational climate–innovative achievements relationship at the job level.

The presented results are of high interest for industrial practitioners, managers, shareholders, and administrators who intend to foster novel ideas and innovation in the workplace. The complex model sheds light on the different elements (i.e., organizational innovation climate, task interdependence, and psychological ownership) that managers should take into account and prioritize to promote innovative behaviors. Especially, the moderated mediated effects reflect the complicated realities that promoting innovation may not be that straightforward. It may remind practitioners to consider several elements and their interactions, from the individual, job, and organizational levels. Among them, it is worth noticing the addition of job characteristics and psychological factors, which are also essential but have often been ignored when considering innovation.

## Theoretical Background and Hypotheses

### Organizational Innovation Climate and Employee Innovative Behavior

Organizational innovation climate is the specific product under a particular environment. More specifically, it is defined as the shared perceptions among employees about the contextual factors that support organizational innovation (van der Vegt et al., 2005). In turn, perceived organizational climate influences their attitudes, values, motivations, commitment, and innovative behaviors, sequentially affecting the organization’s overall innovation performance and innovation capabilities (Madrid et al., 2014; Montani et al., 2014; Newman et al., 2020). Previous studies have demonstrated that good organizational innovation climate enhances employee job satisfaction, job recognition, psychological involvement, and job performance (Madrid et al., 2014; Balkar, 2015).

On the other hand, according to Scott and Bruce (1994), employee innovative behavior is a multi-stage process capturing idea generation, idea promotion, and idea implementation. Instead of the process of forming and generating thoughts or ideas, subsequent research on employee innovative behavior puts more emphasis on the effects and contribution of innovative behavior to the organization. Innovative behavior benefits both individuals and organizations. Indeed, interactions between individuals and individuals’ interactions with multiple key aspects of the organization have been emphasized in the literature (e.g., Hunter et al., 2007; Zweber et al., 2016). The organizational culture that engages and motivates employees increases the likelihood of innovative behavior (Liu Y. et al., 2019). Theoretically, Kanter (1988) argued that the rise and fall of employee innovation depend on the overall organizational environment. For example, establishing open information exchange and communication mechanisms, recognizing the promotion and encouragement of innovation from senior leaders, and receiving sufficient resources like funds and time are all conducive to employee innovation. Many scholars have empirically confirmed the positive impact of organizational innovation climate on individual innovation (Shanker et al., 2017; Liu F. et al., 2019). An organizational innovation environment is conspicuously associated with individual creativity and innovation performance (Bharadwaj and Menon, 2000; Litchfield et al., 2015). When employees are encouraged by the organization’s leaders and feel the organization’s support for their innovative behavior, they would show a stronger willingness to innovate and propose more innovative ideas. Accordingly, this study hypothesizes as follows:

Hypothesis 1: The organizational innovation climate is positively related to employee innovative behavior.

### The Mediating Role of Psychological Ownership

Psychological ownership has been defined as a psychological state: people’s sense of ownership of objects (material or immaterial) (Pierce et al., 2003), feeling as if the targets (or part of the targets) being extensions of the self (Pierce et al., 2001). Research and social practice have further confirmed that feeling of ownership affects individual psychology, emotion, and behavior. According to the psychological ownership theory (Pierce et al., 2003; Van Dyne and Pierce, 2004; Avey et al., 2009), self-efficacy, sense of belonging, sense of responsibility, and self-identity are critical elements for constructing psychological ownership. This study argues that the organizational innovation climate can effectively meet the aforementioned psychological needs of employees. First, in an innovation-friendly environment, employees’ novel ideas are supported and rewarded (Hunter et al., 2007; Charbonnier-Voirin et al., 2010; Bibi et al., 2020). Consequently, employees are more likely to view themselves as creative people and think out of the box or propose alternative solutions without fear of failure. In other words, their sense of self-efficacy and self-identity is stimulated, and they are less prone to give up creative efforts under risks (Liu et al., 2016; Zhou et al., 2021). Second, the organizational innovation climate fulfills the employees’ needs for a sense of belonging by encouraging sharing and interacting thoughts and strengthening the psychological connection between employees and the organization (Hammond et al., 2011; Übius et al., 2013). Furthermore, Aslan and Ateşoğlu (2021) defined the job-based dimension of psychological ownership as the feeling developed due to responsibilities and obligations. The perceived creative organizational climate may then imply an increased sense of responsibility in innovation. Similarly, the sense of commitment can also be incurred by the feeling of ownership and organizational support, facilitating employees to engage in innovation in the workplace (Liu F. et al., 2019). With a feeling of psychological ownership, employees become more attached to, protective of, and responsible for their organizations (Pierce et al., 2001, 2003; Van Dyne and Pierce, 2004; Liu et al., 2012), leading to a series of positive impacts on employees, for example, caring more about organizational innovation and improving work efficiency through hard work and innovative behavior (Wagner et al., 2003; Cheng et al., 2021).

Accordingly, organizational innovation climate affects employee innovative behavior indirectly. Internal motivation, self-efficacy, and individual psychological factors are critical mediating factors between organizational innovation climate and innovative behavior (Tierney et al., 1999; Hunter et al., 2007; Hammond et al., 2011; Übius et al., 2013). As a positive psychological emotion generated in the workplace, psychological ownership is characterized by a strong sense of belonging to the organization (Avey et al., 2009) and a sense of responsibility. It inspires employees to devote time and efforts, assume risks, and make personal sacrifices on behalf of the organization (Pierce et al., 2003; Ramos et al., 2014). Interestingly, these and other pro-organizational attitudes, behaviors, and individual-level outcomes have long been recognized as antecedents of individuals’ innovation activities. Dawkins et al. (2017) pointed out that psychological ownership determines the performance level in relation to employees’ commitment, work engagement, and job satisfaction as these can be positively maintained through their psychological attitudes. When the organization itself nurtures innovation climate, employees perceive organizational support and focus on creation. Based on the previous discussion, the following hypothesis is proposed:

Hypothesis 2: Employees’ psychological ownership mediates the relationship between organizational innovation climate and employee innovative behavior.

### The Moderating Role of Task Interdependence

Task interdependence refers to the degree to which employees need to rely on and cooperate with each other to complete a task (van der Vegt et al., 2001). Turner and Lawrence (1965) first incorporated task interdependence into the six characteristics of jobs. They believed that jobs should include interactions to complete the tasks. However, this kind of interaction is not limited to the need for cooperation and communication among organization members (Bachrach et al., 2006; de Dreu, 2007; Hon and Chan, 2013; Rosen et al., 2018) but also reflects the dependence on the organization’s information, resources, and support during work (Crawford and Haaland, 1972; van der Vegt et al., 2003; Bachrach et al., 2006; Courtright et al., 2015). The higher the task interdependence, the higher the employees’ demand for organizational resources, interaction, and cooperation among members (Peng et al., 2019). High task interdependence implies common goals among members, which could facilitate communication, provide more alternative solutions, obtain more information to improve decision-making effectiveness, and in turn, stimulate innovative behavior (Molm, 1994). Studies have shown the positive impact of task interdependence on organizational performance, employee satisfaction, and team creativity (Taggar, 2002; Liu et al., 2017).

Task interdependence as the core job characteristic is a dimension related to employee innovative behavior. Scott and Bruce (1994) hold that support for innovation within the organization, interaction among members, and resources could trigger employee innovation. Compared with working alone, task interdependence generally leads to more team cooperation, helping behavior, and information sharing (Lee et al., 2015; Fong et al., 2018). Moreover, some studies have proved that job characteristics do not directly affect performance but play a moderating role between the cognitive work environment and the effectiveness of business units (Kiggundu, 1983; Campion et al., 1993; Hon and Chan, 2013). By strengthening communication and interaction among members and task interdependence effectively promotes positive emotional attitudes among employees and positively impacts their behavior (Ramamoorthy and Flood, 2004; Courtright et al., 2015). In an organization with high task interdependence, members need to interact and communicate to complete the job. Cooperation strengthens the interdependence among individuals and, thus, promotes the quality of interpersonal interaction and facilitates the generation of individual psychological ownership. On the contrary, when task interdependence is relatively low, employees can work alone, lacking the need for interaction and cooperation, and are prone to fall into a fixed work model of following the prescribed ordering and “rules” and, therefore, impede the generation of psychological ownership. Even if the organization strengthens the construction of innovation climate, low task interdependence weakens employees’ enthusiasm for cooperation and sharing, ultimately reducing their psychological ownership. Therefore, this study argues that task interdependence in organizational job design does not directly affect employees’ innovative behavior but moderates the relationship between organizational innovation climate and employees’ psychological ownership. Thus, this study proposes the hypothesis as follows:

Hypothesis 3: Task interdependence moderates the positive relationship between organizational innovation climate and employees’ psychological ownership.

### The Integrated Moderated Mediator Model

**FIGURE 1:**
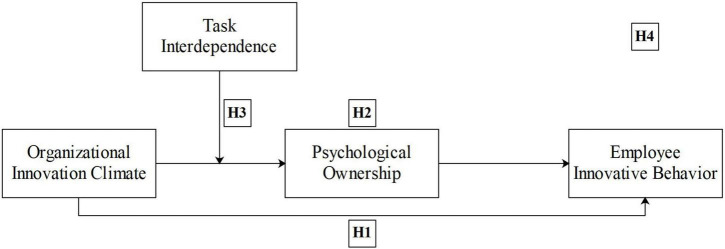
Theoretical model. H4 refers to the moderated mediator model integrating all the elements in this figure.

Together, the aforementioned considerations describe a model in which organizational innovation climate positively relates to employee innovative behavior (Hypothesis 1) and where employee psychological ownership mediates such a positive relationship (Hypothesis 2). However, the strength of the organizational innovation climate and employee psychological ownership depend on task interdependence (Hypothesis 3). These hypotheses together specify a moderated mediator model (Preacher et al., 2007), in which organizational innovation climate is positively but indirectly related to employee innovative behavior through employees’ psychological ownership, with the climate–psychological ownership linkage varying by the level of task interdependence (see Figure 1). As we predict strong (weak) linkages between organizational innovation climate and employee psychological ownership when the task interdependence is high (low), the following is assumed:

Integrating Hypotheses 2 and 3, this study proposes a moderated mediator model. Specifically, the degree of the indirect effect of organizational innovation climate on employee innovative behavior through psychological ownership depends on the level of task interdependence. The higher the level of interdependence at work, the stronger the positive influence of organizational innovation climate on employees’ psychological ownership, which further leads to a greater mediating effect of psychological ownership between organizational innovation climate and employee innovative behavior. Accordingly, we propose the following hypothesis:

Hypothesis 4: The indirect effect of organizational innovation climate on employee innovative behavior through psychological ownership is moderated by task interdependence.

## Materials and Methods

### Sample and Procedures

The sample of this study was employees from headquarters of large internet information enterprises, telecommunication enterprises, joint-stock banks, and a securities company. Large enterprises in these fields tend to dedicate more time and resources to R&D to introduce new products and improve existing ones (e.g., Arbussà and Coenders, 2007). The leader–member paired questionnaires were adopted to minimize the common method bias.

Responses were received through a combination of paper and electronic online surveys. In the paper surveys, department/team leaders were invited by the contact persons in the company and responsible for distributing questionnaires to five to eight direct subordinates. All participants were informed that the survey was only for academic research and assured the confidentiality of their responses. The completed questionnaires were sealed by subordinates and then returned to their supervisors. As for the online surveys, selected team leaders distributed member questionnaires to their subordinates by email. The subordinates were asked to complete their responses for a limited period of time and email them back to their leaders.

A total of 450 pairs of questionnaires were sent out, and 380 pairs were returned. Of these, 326 pairs of valid leader–member questionnaires were obtained with an effective response rate of 72.4%. The team size ranged from five to eight members, and the average number of team members was 4.376. The sample included 206 (63.1%) male and 120 (36.9%) female participants. In terms of age, 36.1% were between the age of 20 and 29 years, 40.2% were between 30 and 39 years, and 23.7% were older than 40 years. In terms of education levels, 37.1% of participants had a master’s degree or above, 54.6% held a bachelor’s degree, and 8.3% had a college degree. For the job level, 58.2% were ordinary employees, 28.3% were first-line managers, and 13.5% were middle managers. Furthermore, the majority of the respondents (56.4%) were from R&D positions.

### Measures

Specifically, team leaders were asked to provide basic information about the team and evaluate five to eight direct subordinates’ innovative behaviors. Team members answered questionnaires about demographics, perceived organizational innovation climate, psychological ownership, and task interdependence. Finally, we constructed such variables as follows:

#### Organizational Innovation Climate

We accessed organizational innovation climate by adapting a 35-item scale from Chiou et al. (2009), which integrated Amabile et al.’s (1996) KEYS (Assessing the Climate for Creativity). It included seven dimensions: organizational philosophy, working methods, resource provision, team operation, leadership effectiveness, learning and growth, and working environment and atmosphere. This scale was measured on a five-point Likert scale ranging from 1 (strongly disagree) to 5 (strongly agree). Totally, eight items with low factor loadings were deleted as a result of the confirmatory factor analysis. Cronbach’s alpha for this scale was 0.874.

#### Employee Innovative Behavior

Employee innovative behavior was measured by a six-item scale from Scott and Bruce (1994). Instead of self-ratings, an employee’s innovative behavior was evaluated by his/her direct leader on a five-point Likert-type scale from 1 (strongly disagree) to 5 (strongly agree). Sample items included “He/She always seeks new theories, techniques and methods” and “He/She often thinks about things from different perspectives.” Cronbach’s alpha of the scale was 0.902.

#### Psychological Ownership

Psychological ownership was assessed using a four-item scale developed by Van Dyne and Pierce (2004). The original scale had seven items. Since one item referred to the mutual sense of ownership and two items were not appropriate for the Chinese culture, only four items were retained to measure psychological ownership. Similar to Peng and Pierce (2015) and Jiang et al. (2019), a five-point Likert scale from 1 (strongly disagree) to 5 (strongly agree) was used to address the Chinese context. Example items included “I feel this is my organization” and “I have a high sense of belonging to the organization.” Cronbach’s alpha for the scale was 0.781.

#### Task Interdependence

Task interdependence was measured by using a three-item scale compiled by Campion et al. (1993), which was a seven-point Likert scale ranging from 1 (completely disagree) to 7 (completely agree). A sample item was “I can’t finish my work without the work information or materials provided by other members.”

#### Control Variables

Empirical studies have shown that demographic variables may significantly affect employee innovative behavior, including gender, age, and education levels (e.g., Van Dyne and Pierce, 2004; Jiang et al., 2019; Liu Y. et al., 2019). Thus, we set these three employee demographics as control variables to ensure the findings hold irrespective of these individual attributes. Gender was measured as a binary variable (1 = male, 2 = female). Age was divided into five levels (1 = less than 25, 2 = 26-35, 3 = 36-45, 4 = 46-55, 5 = greater than 60). The education level was also divided into four categories: high school, college, bachelor, and postgraduate.

### Common Method Bias

Collecting data from the same source (employees) may cause common method bias. Measures were taken to reduce the possible common method bias during data collection, such as using leader–member pairing mode and multiple sources. Specifically, independent variables and dependent variables were collected separately, by having leaders instead of employees report on employee innovative behavior. Furthermore, this study also used Harman’s single-factor test to examine the possible common method deviations. The results showed that the most covariance explained by one factor was 20.7%, less than the cutoff value of 50%. The cumulative can explain 71.40% of the variance. As no single factor explained large variance, common method bias was not a potential problem in our study.

## Results

### Confirmatory Factor Analysis

First, we used Cronbach’s alpha to test the internal consistency (or reliability) of related variables. Cronbach’s alphas of organizational innovation climate, employee innovative behavior, employee psychological ownership, and task interdependence were 0.874, 0.902, 0.781, and 0.815, respectively, all above the recommended value of 0.7, indicating relatively high reliability.

Before testing the hypothesized relationships, we conducted confirmatory factor analysis (CFA) using AMOS 24 to assess the quality of our survey measures. The results of the CFA are presented in Table 1. It shows that the hypothesized four-factor measurement model (i.e., organizational innovation climate, employee innovative behavior, psychological ownership, and task interdependence) provided good fit to the data (χ^2^/df = 3.29, *p* < 0.001; CFI = 0.94; TLI = 0.91; RMSEA = 0.08), which yielded better fit than all alternative three-factor, two-factor, and one-factor models. These findings demonstrated the discriminant validity of the measures of our focal constructs.

**TABLE 1:** Confirmatory factor analysis results.

Model	χ^2^	*df*	χ^2^/*df*	CFI	TLI	RMSEA	CI 90% RMSEA
Four-factor model	516.53	157	3.29	0.94	0.91	0.08	[0.073, 0.081]
OIC + PO, TI, EIB	859.36	164	5.24	0.80	0.83	0.11	[0.103, 0.118]
OIC, PO + TI, EIB	894.66	162	5.52	0.89	0.80	0.13	[0.130, 0.136]
OIC, PO, TI + EIB	982.54	162	6.07	0.88	0.78	0.15	[0.147, 0.152]
OIC + PO + TI, EIB	1093.98	168	6.51	0.87	0.77	0.18	[0.175, 0.181]
One-factor model	1561.67	172	9.08	0.80	0.65	0.20	[0.201, 0.217]

*N = 326. OIC, organizational innovation climate; PO, psychological ownership; EIB, employee innovative behavior; TI, task interdependence. CFI, (Bentler’s) comparative fit index; TLI, Tucker–Lewis index; RMSEA, root mean square error of approximation; CI, confidence interval.*

### Descriptive Statistics

The means, standard deviations, and correlation coefficients of all variables are given in Table 2. As expected, organizational innovation climate was positively associated with psychological ownership (*r* = 0.70; *p* < 0.05) and employee innovative behavior (*r* = 0.68; *p* < 0.05). Psychological ownership was positively related to innovative behavior (*r* = 0.57; *p* < 0.05). All the correlation coefficients did not exceed 0.70, indicating no obvious collinearity problem among the variables. The results provided preliminary support for Hypotheses 1 and 2.

**TABLE 2:** Descriptive statistics and correlations of variables.

Variable	*M*	*SD*	1	2	3	4	5	6	7
1 Gender	1.27	0.45	–						
2 Age	2.09	0.33	0.16	–					
3 Education level	3.07	0.53	0.06	0.12*	–				
4 OIC	3.59	0.27	0.17	0.33	0.10	–			
5 PO	3.05	0.43	0.19**	0.32	0.08	0.70**	–		
6 TI	3.03	0.65	0.20	0.07	0.12*	0.29**	0.36**	–	
7 EIB	3.59	0.72	0.28**	0.02	0.08	0.68**	0.57**	0.22**	–

N = 326. *p < 0.05, **p < 0.01. OIC, organizational innovation climate; EIB, employee innovative behavior; PO, psychological ownership; TI, task interdependence. For gender, 1 = “male” and 2 = “female.” For age, 1 = “less than 25,” 2 = “26–35,” 3 = “36–45,” 4 = “46–55,”and 5 = “greater than 60.” For education level, 1 = “high school and below,” 2 = “college degree,” 3 = “bachelor degree,” and 4 = “postgraduate (Master/PhD)’s degree.”

### Hypothesis Tests

We conducted hierarchical regression analysis to test the hypotheses (see Table 3). We first entered all control variables into the model (M3) and then added organizational innovation climate into the model (M4). After controlling for employee gender, age, and education level, organizational innovation climate was significantly related to employee innovative behavior (M4, β = 0.455, *p* < 0.001), and therefore, Hypothesis 1 was supported.

**TABLE 3:** Hierarchical regression analysis results.

Variables	PO		EIB				PO
	M1	M2	M3	M4	M5	M6	M7
Gender	0.151	0.068	0.289	0.196	0.199	0.184	0.120*
Age	–0.312	–0.088	0.013	0.266	0.198	0.281	−0.135*
Education level	–0.126	–0.027	–0.095	0.017	–0.020	0.021	–0.082
OIC		0.570***		0.455***		0.342	0.547*
PO					0.595***	0.570***	
TI							0.240*
OIC × TI							0.212***
*F*	17.379	87.306***	10.454***	108.369***	51.980***	91.453***	71.025***
*R* ^2^	0.139	0.521	0.089	0.575	0.393	0.588	0.572
Adjusted *R*^2^	0.131	0.515	0.080	0.569	0.386	0.582	0.564
Δ*R^2^*	−	0.382	−	0.486	0.304	0.500	0.051

N = 326; *p < 0.05, ***p < 0.001. OIC, organizational innovation climate; PO, psychological ownership; TI, task interdependence; EIB, employee innovative behavior.

Second, to test Hypothesis 2 in an integrated fashion, we performed a bootstrapping procedure with the SPSS PROCESS macro (Hayes, 2013). After considering control variables, organizational innovation climate was significantly associated with employee psychological ownership (M2, β = 0.570, *p* < 0.001), and psychological ownership was significantly related to employee innovative behavior (M5, β = 0.595, *p* < 0.001). Then, organizational innovation climate and psychological ownership were entered into the regression model simultaneously. The significant association between psychological ownership and employee innovative behavior still existed (M6, β = 0.570, *p* < 0.001), but the significant relationship between organizational innovation climate and employee innovative behavior (M4, β = 0.455, *p* < 0.001) became insignificant (M6, β = 0.342, *p* > 0.05). This result suggested that psychological ownership played a full mediating role in the relationship between organizational innovation climate and employee innovative behavior. Thus, Hypothesis 2 was supported.

Third, we tested the moderating role of task interdependence in the relationship between organizational innovation climate and employee psychological ownership. As shown in M7, the interaction of organizational innovation climate and task interdependence was incorporated into the regression model. The interaction effect was significant and positive (M7, β = 0.212, *p* < 0.001), indicating that task interdependence moderated the relationship between organizational innovation climate and psychological ownership. The result provided evidence for Hypothesis 3.

**FIGURE 2:**
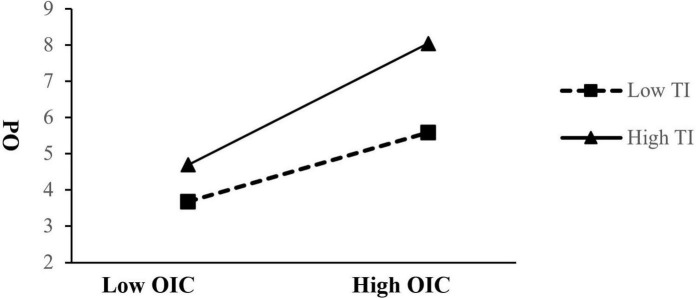
The moderating effect of task interdependence.

To further illustrate the moderating effect (Aiken et al., 1991), we plotted the relationship between organizational innovation climate and psychological ownership for low (1 *SD* less than the mean) and high (1 *SD* greater than the mean) task interdependence in Figure 2. The form of the interaction corroborated the predicted pattern, with the linkage between organizational innovation climate and psychological ownership being more pronounced for those with high, rather than low, task interdependence. Again, it supported Hypothesis 3.

To test Hypothesis 4 (moderated mediation) in an integrated fashion, we used the PROCESS macro for SPSS (Hayes, 2013). We first entered gender, age, and education level values as controls; organizational innovation climate as the predictor; psychological ownership as the mediator; task interdependence as the first-stage moderator; and employee innovative behavior as the dependent variable. Next, in accordance with Jiang et al.’s (2019) study, we set the bootstrap sample to 5,000 and chose the “Mean center for construction of products” in options, by which used variables can be automatically mean-centered prior to the construction of products. Table 4 depicts the results of the conditional indirect relationship between organizational innovation climate and employee innovative behavior through employee psychological ownership at different values of task interdependence. Specifically, when the degree of task interdependence is low (1 *SD* less than the mean), the indirect effect is significant [bootstrapping indirect effect = 0.16, *SE* = 0.08, 95% CI (0.11, 0.42), excluding 0]. When the degree of task interdependence is high (1 *SD* greater than the mean), the indirect effect is also significant [bootstrapping indirect effect = 0.31, *SE* = 0.08, 95% CI (0.08, 0.38), excluding 0] and the index of moderated mediation is significant [moderated mediation index = 0.26, *SE* = 0.06, 95% CI (0.12, 0.38)], suggesting that the strength of two conditional indirect effects was significantly different. Thus, Hypothesis 4 was supported, indicating that when task interdependence is of a higher level, organizational innovation climate has a stronger relation with employee innovative behavior through psychological ownership.

**TABLE 4:** Conditional indirect effect of OIC on EIB through PO at different values of TI.

TI	OIC → PO → EIB			
	Boot indirect effect	Boot SE	Boot LLCI	Boot ULCI
−1 SD	0.16	0.08	0.11	0.42
Mean	0.24	0.07	0.10	0.40
+1 SD	0.31	0.08	0.08	0.38

*Controlling for gender, age, and education level. SE, standard error; -1 SD, one standard deviation less than the mean value of TI; Mean, mean value of TI; +1 SD, one standard deviation greater than the mean value of TI. Bootstrap n = 5,000. OIC, organizational innovation climate; PO, psychological ownership; TI, task interdependence; EIB, employee innovative behavior; LLCI, lower limit confidence interval; ULCI, upper limit confidence interval.*

## Discussion

All elements included within the tested model have been found to affect employee innovative behaviors. The results show that organizational innovation climate is positively related to employee innovative behavior, which is congruent with Amabile and Gryskiewicz’s (1989) model of creativity and innovation in the organization and most research on the relations between organizational innovation climate and employee innovation-related outcomes (e.g., Hunter et al., 2007; Hammond et al., 2011; Übius et al., 2013; Liu F. et al., 2019; Olsson et al., 2019).

In particular, this study introduces psychological ownership into the exploration of organizational innovation climate and employee innovative behavior. Although scholars, such as Liu F. et al. (2019), have examined the positive link between psychological ownership and innovative behavior, this study focuses on its indirect mediating role. The results reveal a significant indirect effect of organizational innovation climate on employee innovative behavior through psychological ownership. Yet, these results are also in contrast with those using psychological ownership as the mediating variable. The possibility of using it as the mediating variable has been supported in other relationships, such as between affective commitment and knowledge sharing (Li et al., 2015), between empowering leadership and organizational citizenship behavior (Jiang et al., 2019), between innovation and growth opportunities (Santoso, 2020), and between creativity and knowledge creation (Yoon et al., 2020). Indeed, to our knowledge, how employee psychological ownership mediates the relationship between organizational innovation climate and employee innovative behaviors has not been explored previously.

Compared with other job characteristics, task interdependence has been underexamined in the conversation related to innovative behaviors. Specifically, considering the high volume of cooperation and trade within and between employees, organizations, sectors, and even nations nowadays, it is vital to address and examine such job characteristics. A considerable amount of literature has been published on the moderating role of task interdependence in other relationships, such as between helping behavior and group performance (Bachrach et al., 2006), in the direct effects of empowering leadership on team self-concordance and team creative efficacy (Hon and Chan, 2013); between leader humility and team innovation (Liu et al., 2017); between knowledge hiding and team creativity (Fong et al., 2018); and between empowering leadership concerning team meaningfulness (Lisak et al., 2022). Nevertheless, its impact on psychological ownership has been poorly understood. This study illustrates that task interdependence positively moderates the relationship between organizational innovation climate and psychological ownership, enriching the psychological formation mechanism of organizational innovation climate.

Connecting factors at the job level and from the psychological perspective, our study attempts to put all these variables into one single complex model, which also allows for the exploration of possible moderating and mediating effects. The higher the degree of task interdependence at work, the stronger the mediating effect of psychological ownership between organizational innovation climate and employee innovative behavior, and vice versa. Taken together, the study supported all the proposed hypotheses.

### Theoretical Implications

In a recent review, Anderson et al. (2018) called for more research to better understand individual innovation in organizational and managerial practices in different countries. The main theoretical contribution of this study is to draw attention to the relationship between organizational innovation climate and innovative behavior as an indirect relationship through psychological ownership, moderated by task interdependence. The results demonstrate a more complicated relationship between the innovation climate in the organization and its effects on individuals.

First, the results confirm the positive relationship between organizational innovation climate and employee innovative behavior, which is consistent with previous studies (e.g., Kanter, 1988; Shanker et al., 2017; Liu F. et al., 2019; Bibi et al., 2020). This study adds to existing knowledge by examining such a relationship with the employees in industries where product and service innovation are highly valued. These jobs require employees to innovate. Since higher demand for creativity generally leads to more innovative behaviors (Robinson and Beesley, 2010), we expect to see more significant effects of innovation in these industries. Finance, telecommunication, software, and information technology services are among our target populations to mitigate the inherent variation in demand for innovation across industries. Moreover, we adopted the leader–member paired questionnaires to attenuate the common method bias, with employees assessing the organizational innovation climate and their leaders evaluating the employees’ innovative behavior.

Second, the present study draws on employees’ psychological theory to clarify how organizational innovation climate promotes employee innovative behavior. Although psychological factors have been considered as important predictors of employee innovation, little empirical research is available. Recently, Liu F. et al. (2019) used psychological ownership as a moderator of the relationship between organizational innovation climate and employee innovative behavior but failed to claim a significant effect. However, our study confirms the mediating role of psychological ownership, supplementing the psychological formation of employee innovative behavior motivated by organizational innovation climate.

Finally, the relationship between job characteristics and employee innovative behaviors has seldom been the focus of researchers’ attention (Shalley and Gilson, 2004). In an attempt to fill this gap, this study shows that task interdependence acts as a synergistic effect interacting with organizational innovation climate on psychological ownership. According to the job characteristics theory by Hackman and Oldham (1976), job dimensions (i.e., skill variety, task identity, task significance, autonomy, and feedback) stimulate experienced meaningfulness, responsibility, and knowledge of the results of the work, that is, job characteristics affect one’s psychological states, which in turn change personal and work outcomes. Likewise, high task interdependence is frequently related to increased communication, help, information sharing, and other forms of cooperation (Bachrach et al., 2006; Fong et al., 2018). These two viewpoints are supported simultaneously in this study: when task interdependence is strong, organization members are expected to work through interaction and communication in response to the need for cooperation, promoting interdependence among members; meanwhile, the construction of organizational innovation climate can enhance willingness to share and interdependence, promote cohesion within the organization, and increase employees’ sense of belonging and sense of responsibility to the organization, thus fostering more individual innovative behavior under interdependent tasks than under individualistic ones. Taking task interdependence as a moderating variable, this study responds to Hunter et al.’s (2007) calling for the importance of paying more attention to the moderators in the relationship between climate and innovative achievements from the job level.

### Managerial Implications

The results of our study also offer practical implications for organizations and managers that value employees’ innovative behavior. First, this study shows that organizational innovation climate can increase employees’ psychological ownership and drive them to engage in innovation. Accordingly, managers can motivate psychological ownership and innovative behavior by consolidating the organizational innovation climate, such as setting innovation working goals and developing an open platform to guarantee support for innovative behaviors.

Second, the psychological ownership of non-managerial employees is an important antecedent of innovative behavior (Leyer et al., 2021). In the present study, we found that psychological ownership played a mediating role in the relationship between organizational innovation climate and employee innovative behavior. Thus, organizations can strengthen employees’ possessive feelings, perception of encouragement, and attention to innovation from corporate culture and management practice. When employees regard innovation as their own responsibility and mission, they will consciously devote themselves to innovation and actively seek creative solutions.

Third, in the process of motivating employee innovative behavior, job characteristics should not be overlooked (Hackman and Oldham, 1976; Hunter et al., 2007). Innovation is not simple, repetitive work. It often requires breaking routines and changing the inherent way of working. While individual knowledge is limited, the interaction and even collision among members can induce unexpected inspirations, thereby invigorating employees and stimulating innovation. By building a free and open communication and learning platform, employees of different positions and hierarchies can easily seek help or share innovation progress. Gradually, interaction and cooperation are promoted, and thereby improving innovation efficiency. In addition, human resource management practitioners could also redesign the job tasks to enhance the connection among organization members, promote the free flow of information among different departments and positions, and provide more support for innovative behavior.

### Limitations and Suggestions for Future Research

There are several limitations worth noting. First, this study utilized a cross-sectional design. Since the data were collected at about the same time, we cannot investigate the dynamic causality in addition to the correlation. Future research could use longitudinal or experiment data to explore the causal relations between the model variables.

Second, the relatively small sample size might raise concerns about the generalizability and robustness of the research findings. To alleviate the common method bias, we collected data with the leader–member paired questionnaires, that is, in addition to employees’ self-reports, the leaders were asked to evaluate their employees’ innovative behaviors. However, the use of matched samples limited our ability to gather more valid responses from the headquarters of Guangdong to some extent. In the future, researchers could consider expanding the study by involving employees and leaders in other provinces or nations.

Third, surveys are essentially subjective. Future studies could utilize objective proxies, such as using the number of patents, to gauge innovative behavior and conducting textual analysis on corporate social responsibility to reflect organizational innovation climate.

Fourth, while there is merit in using task interdependence as a tool in an innovation model to explore the possible moderating role of job characteristics, our choice of interdependence remains task-centric which may not evolve with the changing world. According to Raveendran et al. (2020), interdependence in an organization’s workflows should be at least three-fold: task, goal, and knowledge. Therefore, there is abundant room for further progress in testing possible differential dynamics between moods and specific aspects of interdependence and other job characteristics. Similarly, Pierce and Jussila (2010) proposed that psychological ownership can emerge at both the individual and group levels. Future studies can explore the mechanism with such collective psychological ownership. Moreover, more recent measures of task interdependence, for example, can be used to address the possible change in the construct.

## Conclusion

Employee innovative behavior is critical to achieving sustainable competitive advantages. In the present study, the mechanism underlying employee innovative behavior is investigated at the organizational, individual, and job levels. The achieved results do not only confirm some prior research studies on the positive role of organizational innovation climate on employee innovative behavior (e.g., Shanker et al., 2017; Liu F. et al., 2019) but also extend them by underlining the indispensable role of psychological ownership and task interdependence, which has seldom emerged in those studies.

The results show a moderated mediating relationship. In specific, psychological ownership mediates the positive relationship between organizational innovation climate and employee innovative behavior, while task interdependence fosters both employee psychological ownership and innovative behavior, acting as a complement to the organizational innovation climate. Notwithstanding some limitations, our study offers valuable empirical evidence that can raise the theoretical and practical debate on how to promote employee innovative behaviors.

## Data Availability Statement

The datasets generated for this study are available from the corresponding author on reasonable request.

## Ethics Statement

The studies involving human participants were reviewed and approved by the Ethics Committee of South China Normal University and Guangdong Industry Polytechnic. Written informed consent was obtained from all participants for their participation in this study.

## Author Contributions

YY contributed to the conception, data curation, formal analysis, and manuscript writing and editing. ZH contributed to the conception and revision of the manuscript. JL contributed to the review and revision of the manuscript. YW contributed to the editing of the manuscript. MX contributed to the editing and revision of the manuscript. All authors contributed to the article and approved the submitted version.

## Conflict of Interest

The authors declare that the research was conducted in the absence of any commercial or financial relationships that could be construed as a potential conflict of interest.

## Publisher’s Note

All claims expressed in this article are solely those of the authors and do not necessarily represent those of their affiliated organizations, or those of the publisher, the editors and the reviewers. Any product that may be evaluated in this article, or claim that may be made by its manufacturer, is not guaranteed or endorsed by the publisher.

## References

[B1] Abbas A. L. Ye C. Zhuo S. Manzoor S. Ullah I. (2022). Role of responsible leadership for organizational citizenship behavior for the environment in light of psychological ownership and employee environmental commitment: a moderated mediation model. *Front. Psychol.* 12:13. 10.3389/fpsyg.2021.756570 35211051 PMC8862681

[B2] Aiken L. S. West S. G. Reno R. R. (1991). *Multiple Regression: Testing and Interpreting Interactions.* Thousand Oaks, CA: Sage.

[B3] Amabile T. M. Conti R. Coon H. Lazenby J. Herron M. (1996). Assessing the work environment for creativity. *Acad. Manage. J.* 39 1154–1184. 10.5465/256995 256995

[B4] Amabile T. M. Gryskiewicz N. D. (1989). The creative environment scales: work environment inventory. *Creativ. Res. J.* 2 231–253. 10.1080/10400418909534321

[B5] Anderson N. Potočnik K. Bledow R. Hulsheger U. Rosing K. (2018). “Innovation and creativity in organizations,” in *Handbook of Industrial, Work and Organizational Psychology*, eds Ones D. Anderson N. Viswesvaran C. Sinangil H. K. (Thousand Oaks, CA: Sage).

[B6] Arbussà A. Coenders G. (2007). Innovation activities, use of appropriation instruments and absorptive capacity: evidence from Spanish firms. *Res. Policy* 36 1545–1558. 10.1016/j.respol.2007.04.013

[B7] Aslan M. Ateşoğlu H. (2021). Psikolojik sahiplenme ölçeğinin türkçe uyarlamasi, güvenilirlik ve geçerlilik Çalişmalari (turkish adaptation, reliability and validity studies of psychological ownership scale). işletme araştirmalari dergisi. *J. Bus. Res. Turk* 12 4184–4195. 10.20491/isarder.2020.1098

[B8] Avey J. B. Avolio B. J. Crossley C. D. Luthans F. (2009). Psychological ownership: theoretical extensions, measurement and relation to work outcomes. *J. Organ. Behav.* 30 173–191. 10.1002/job.583

[B9] Avey J. B. Wernsing T. S. Palanski M. E. (2012). Exploring the process of ethical leadership: the mediating role of employee voice and psychological ownership. *J. Bus. Ethics* 107 21–34. 10.1007/s10551-012-1298-2

[B10] Awang A. Sapie N. M. Hussain M. Y. Ishal S. Yusof R. M. (2019). Nurturing innovative employees: effects of organisational learning and work environment. *Ekon. Istraz.* 32 1152–1168. 10.1080/1331677x.2019.1592007

[B11] Bachrach D. G. Powell B. C. Collins B. J. Richey R. G. (2006). Effects of task interdependence on the relationship between helping behavior and group performance. *J. Appl. Psychol.* 91 1396–1405. 10.1037/0021-9010.91.6.1396 17100493

[B12] Balkar B. (2015). The relationships between organizational climate, innovative behavior and job performance of teachers. *Int. Online J. Educ. Sci.* 2015:7. 10.15345/iojes.2015.02.007

[B13] Bharadwaj S. Menon A. (2000). Making innovation happen in organizations: individual creativity mechanisms, organizational creativity mechanisms or both? *J. Prod. Innov. Manage.* 17 424–434. 10.1111/1540-5885.1760424

[B14] Bibi S. Khan A. Qian H. D. Garavelli A. C. Natalicchio A. Capolupo P. (2020). Innovative climate, a determinant of competitiveness and business performance in Chinese law firms: the role of firm size and age. *Sustainability* 12:24. 10.3390/su12124948

[B15] Campion M. A. Medsker G. J. Higgs A. C. (1993). Relations between work group characteristics and effectiveness: implications for designing effective work groups. *Pers. Psychol.* 46 823–847. 10.1111/j.1744-6570.1993.tb01571.x

[B16] Charbonnier-Voirin A. El Akremi A. Vandenberghe C. (2010). A multilevel model of transformational leadership and adaptive performance and the moderating role of climate for innovation. *Group Organ. Manage.* 35 699–726. 10.1177/1059601110390833

[B17] Cheng Z. H. Liu W. X. Zhou K. Che Y. J. Han Y. (2021). Promoting employees’ pro-environmental behaviour through empowering leadership: the roles of psychological ownership, empowerment role identity, and environmental self-identity. *Bus. Ethics Environ. Responsib.* 30 604–618. 10.1111/beer.12366

[B18] Chiou H.-J. Chen Y.-J. Lin P.-F. (2009). Development of creative organizational climate inventory and validation study. *Psychol. Test.* 56 69–97. 10.7108/pt.200903.0069

[B19] Courtright S. H. Thurgood G. R. Stewart G. L. Pierotti A. J. (2015). Structural interdependence in teams: an integrative framework and meta-analysis. *J. Appl. Psychol.* 100 1825–1846. 10.1037/apl0000027 25938722

[B20] Crawford J. L. Haaland G. A. (1972). Predecisional information seeking and subsequent conformity in the social influence process. *J. Pers. Soc. Psychol.* 23 112–119. 10.1037/h0032870

[B21] Cristofaro M. (2021). Organizational sensemaking: a systematic review and a co-evolutionary model. *Eur. Manag. J.* 10.1016/j.emj.2021.07.003 [Epub ahead of print].

[B22] Dawkins S. Tian A. W. Newman A. Martin A. (2017). Psychological ownership: a review and research agenda. *J. Organ. Behav.* 38 163–183. 10.1002/job.2057

[B23] de Dreu C. K. W. (2007). Cooperative outcome interdependence, task reflexivity, and team effectiveness: a motivated information processing perspective. *J. Appl. Psychol.* 92 628–638. 10.1037/0021-9010.92.3.628 17484546

[B24] Fong P. S. W. Men C. H. Luo J. L. Jia R. Q. (2018). Knowledge hiding and team creativity: the contingent role of task interdependence. *Manag. Decis.* 56 329–343. 10.1108/md-11-2016-0778

[B25] Hackman J. R. Oldham G. R. (1976). Motivation through the design of work: test of a theory. *Organ. Behav. Hum. Perform.* 16 250–279. 10.1016/0030-5073(76)90016-7

[B26] Hammond M. M. Neff N. L. Farr J. L. Schwall A. R. Zhao X. Y. (2011). Predictors of individual-level innovation at work: a meta-analysis. *Psychol. Aesthet. Creat. Arts.* 5 90–105. 10.1037/a0018556

[B27] Hayes A. F. (2013). *Introduction to Mediation, Moderation, and Conditional Process Analysis: A Regression-Based Approach.* New York, NY: The Guilford Press.

[B28] Hon A. H. Y. Chan W. W. H. (2013). Team creative performance: the roles of empowering leadership, creative-related motivation, and task interdependence. *Cornell Hosp. Q.* 54 199–210. 10.1177/1938965512455859

[B29] Hunter S. T. Bedell K. E. Mumford M. D. (2007). Climate for creativity: a quantitative review. *Creativ. Res. J.* 19 69–90. 10.1080/10400410709336883

[B30] Jaiswal N. K. Dhar R. L. (2015). Transformational leadership, innovation climate, creative self-efficacy and employee creativity: a multilevel study. *Int. J. Hosp. Manag.* 51 30–41. 10.1016/j.ijhm.2015.07.002

[B31] Jiang M. Wang H. Li M. (2019). Linking empowering leadership and organizational citizenship behavior toward environment: the role of psychological ownership and future time perspective. *Front. Psychol.* 10:2612. 10.3389/fpsyg.2019.02612 31849746 PMC6895145

[B32] Kanter R. M. (1988). “When a thousand flowers bloom: structural, collective, and social conditions for innovation in organization,” in *Research in Organizational Behavior*, eds Staw B. M. Cummings L. L. (Greenwich: J.A.I. Press).

[B33] Kaplan S. (2008). Framing contests: strategy making under uncertainty. *Organ Sci.* 19 729–752. 10.1287/orsc.1070.0340 19642375

[B34] Kiggundu M. N. (1983). Task interdependence and job design: test of a theory. *Organ. Behav. Hum. Perform.* 31 145–172. 10.1016/0030-5073(83)90118-6 10259647

[B35] Lee C. C. Lin Y. H. Huan H. C. Huang W. W. Teng H. H. (2015). The effects of task interdependence, team cooperation, and team conflict on job performance. *Soc. Behav. Pers.* 43 529–536. 10.2224/sbp.2015.43.4.529

[B36] Lee L. Wong P. K. Foo M. D. Leung A. (2011). Entrepreneurial intentions: the influence of organizational and individual factors. *J. Bus. Ventur.* 26 124–136. 10.1016/j.jbusvent.2009.04.003

[B37] Leyer M. Hirzel A.-K. Moormann J. (2021). It’s mine, I decide what to change: the role of psychological ownership in employees’ process innovation behaviour. *Int. J. Innov. Mgt.* 25:2150013. 10.1142/S1363919621500134

[B38] Li J. Yuan L. Ning L. T. Li-Ying J. (2015). Knowledge sharing and affective commitment: the mediating role of psychological ownership. *J. Knowl. Manag.* 19 1146–1166. 10.1108/jkm-01-2015-0043

[B39] Lisak A. Harush R. Icekson T. Harel S. (2022). Team interdependence as a substitute for empowering leadership contribution to team meaningfulness and performance. *Front. Psychol.* 13:637822. 10.3389/fpsyg.2022.637822 35222170 PMC8879840

[B40] Litchfield R. C. Ford C. M. Gentry R. J. (2015). Linking individual creativity to organizational innovation. *J. Creat. Behav.* 49 279–294. 10.1002/jocb.65

[B41] Liu D. Chen X. P. Yao X. (2011). From autonomy to creativity: a multilevel investigation of the mediating role of harmonious passion. *J. Appl. Psychol.* 96 294–309. 10.1037/a0021294 21058804

[B42] Liu D. Jiang K. F. Shalley C. E. Keem S. Zhou J. (2016). Motivational mechanisms of employee creativity: a meta-analytic examination and theoretical extension of the creativity literature. *Organ. Behav. Hum. Decis. Process.* 137 236–263. 10.1016/j.obhdp.2016.08.001

[B43] Liu F. Chow I. H.-S. Zhang J.-C. Huang M. (2019). Organizational innovation climate and individual innovative behavior: exploring the moderating effects of psychological ownership and psychological empowerment. *Rev. Manag. Sci.* 13 771–789. 10.1007/s11846-017-0263-y

[B44] Liu J. Wang H. Hui C. Lee C. (2012). Psychological ownership: how having control matters. *J. Manage. Stud.* 49 869–895. 10.1111/j.1467-6486.2011.01028.x

[B45] Liu W. Mao J. Chen X. (2017). Leader humility and team innovation: investigating the substituting role of task interdependence and the mediating role of team voice climate. *Front. Psychol.* 8:1115. 10.3389/fpsyg.2017.01115 28713316 PMC5492832

[B46] Liu Y. Wang W. Chen D. (2019). Linking ambidextrous organizational culture to innovative behavior: a moderated mediation model of psychological empowerment and transformational leadership. *Front. Psychol.* 10:2192. 10.3389/fpsyg.2019.02192 31681063 PMC6798063

[B47] Luo Y. Cao Z. Yin L. Zhang H. Wang Z. (2018). Relationship between extraversion and employees’ innovative behavior and moderating effect of organizational innovative climate. *NeuroQuantology* 16 186–194. 10.14704/nq.2018.16.6.1604

[B48] Madrid H. P. Patterson M. G. Birdi K. S. Leiva P. I. Kausel E. E. (2014). The role of weekly high-activated positive mood, context, and personality in innovative work behavior: a multilevel and interactional model. *J. Organ. Behav.* 35 234–256. 10.1002/job.1867

[B49] Mathieu J. E. Hollenbeck J. R. Van Knippenberg D. Ilgen D. R. (2017). A century of work teams in the journal of applied psychology. *J. Appl. Psychol.* 102 452–467. 10.1037/apl0000128 28150984

[B50] Molm L. D. (1994). Dependence and risk: transforming the structure of social exchange. *Soc. Psychol. Q.* 57 163–176. 10.2307/2786874

[B51] Montani F. Odoardi C. Battistelli A. (2014). Individual and contextual determinants of innovative work behaviour: proactive goal generation matters. *J. Occup. Organ. Psychol.* 87 645–670. 10.1111/joop.12066

[B52] Newman A. Round H. Wang S. L. Mount M. (2020). Innovation climate: a systematic review of the literature and agenda for future research. *J. Occup. Organ. Psychol.* 93 73–109. 10.1111/joop.12283

[B53] Olsson A. Paredes K. M. B. Johansson U. Roese M. O. Ritzen S. (2019). Organizational climate for innovation and creativity - a study in Swedish retail organizations. *Int. Rev. Retail Distrib. Consum. Res.* 29 243–261. 10.1080/09593969.2019.1598470

[B54] Peng H. Pierce J. (2015). Job- and organization-based psychological ownership: relationship and outcomes. *J. Manage. Psychol.* 30 151–168. 10.1108/JMP-07-2012-0201

[B55] Peng J. Wang Z. Chen X. (2019). Does self-serving leadership hinder team creativity? A moderated dual-path model. *J. Bus. Ethics* 159 419–433. 10.1007/s10551-018-3799-0

[B56] Phung V. D. Hawryszkiewycz I. Binsawad M. (2018). “Exploring how environmental and personal factors influence knowledge sharing behavior leads to innovative work behavior,” in *Advances in Information Systems Development. Methods, Tools and Management. Lecture Notes in Information Systems and Organisation*, eds Paspallis N. Raspopoulos M. Barry C. Lang M. Linger H. Schneider C. (Cham: Springer), 10.1007/978-3-319-74817-7_7

[B57] Pierce J. L. Jussila I. (2010). Collective psychological ownership within the work and organizational context: construct introduction and elaboration. *J. Organ. Behav.* 31 810–834. 10.1002/job.628

[B58] Pierce J. L. Kostova T. Dirks K. T. (2001). Toward a theory of psychological ownership in organizations. *Acad. Manage. Rev.* 26 298–310. 10.5465/amr.2001.4378028

[B59] Pierce J. L. Kostova T. Dirks K. T. (2003). The state of psychological ownership: integrating and extending a century of research. *Rev. Gen. Psychol.* 7 84–107. 10.1037/1089-2680.7.1.84

[B60] Pieterse A. N. Van Knippenberg D. Schippers M. Stam D. (2010). Transformational and transactional leadership and innovative behavior: the moderating role of psychological empowerment. *J. Organ. Behav.* 31 609–623. 10.1002/job.650

[B61] Preacher K. J. Rucker D. D. Hayes A. F. (2007). Addressing moderated mediation hypotheses: theory, methods, and prescriptions. *Multivariate Behav. Res.* 42 185–227. 10.1080/00273170701341316 26821081

[B62] Ramamoorthy N. Flood P. C. (2004). Individualism/collectivism, perceived task interdependence and teamwork attitudes among Irish blue-collar employees: a test of the main and moderating effects? *Hum. Relat.* 57 347–366. 10.1177/0018726704043274

[B63] Ramos H. M. Man T. W. Y. Mustafa M. Ng Z. Z. (2014). Psychological ownership in small family firms: family and non-family employees’ work attitudes and behaviours. *J. Fam. Bus. Strateg.* 5 300–311. 10.1016/j.jfbs.2014.04.001

[B64] Raveendran M. Silvestri L. Gulati R. (2020). The role of interdependence in the micro-foundations of organization design: task, goal, and knowledge interdependence. *Acad. Manag. Ann.* 14 828–868. 10.5465/annals.2018.0015

[B65] Robinson R. N. S. Beesley L. G. (2010). Linkages between creativity and intention to quit: an occupational study of chefs. *Tourism Manage.* 31 765–776. 10.1016/j.tourman.2009.08.003

[B66] Rosen M. A. Diazgranados D. Dietz A. S. Benishek L. E. Thompson D. Pronovost P. J. (2018). Teamwork in healthcare: key discoveries enabling safer, high-quality care. *Am. Psychol.* 73 433–450. 10.1037/amp0000298 29792459 PMC6361117

[B67] Santoso A. (2020). Impact of psychological ownership on innovation and growth in Indonesia business firms. *Int. J. Psychosoc. Rehabil.* 24 1002–1012. 10.37200/IJPR/V24I7/PR270097

[B68] Schulte M. Ostroff C. Kinicki A. J. (2006). Organizational climate systems and psychological climate perceptions: a cross-level study of climate-satisfaction relationships. *J. Occup. Organ. Psychol.* 79 645–671. 10.1348/096317905x72119

[B69] Scott S. G. Bruce R. A. (1994). Determinants of innovative behavior: a path model of individual innovation in the workplace. *Acad. Manage. J.* 37 580–607. 10.5465/256701 256701

[B70] Shalley C. E. Gilson L. L. (2004). What leaders need to know: a review of social and contextual factors that can foster or hinder creativity. *Leadersh. Q.* 15 33–53. 10.1016/j.leaqua.2003.12.004

[B71] Shanker R. Bhanugopan R. Van Der Heijden B. Farrell M. (2017). Organizational climate for innovation and organizational performance: the mediating effect of innovative work behavior. *J. Vocat. Behav.* 100 67–77. 10.1016/j.jvb.2017.02.004

[B72] Taggar S. (2002). Individual creativity and group ability to utilize individual creative resources: a multilevel model. *Acad. Manage. J.* 45 315–330. 10.2307/3069349

[B73] Tierney P. Farmer S. M. Graen G. B. (1999). An examination of leadership and employee creativity: the relevance of traits and relationships. *Pers. Psychol.* 52 591–620. 10.1111/j.1744-6570.1999.tb00173.x

[B74] Turner A. N. Lawrence P. R. (1965). *Industrial Jobs and the Worker: An Investigation of Response to Task Attributes.* Boston, MA: Harvard University.

[B75] Übius Ü Alas R. Elenurm T. (2013). Impact of innovation climate on individual and organisational level factors in Asia and Europe. *J. Bus. Econ. Manag.* 14 1–21. 10.3846/16111699.2011.642081

[B76] van der Vegt G. S. Emans B. J. M. van de Vliert E. (2001). Patterns of interdependence in work teams: a two-level investigation of the relations with job and team satisfaction. *Pers. Psychol.* 54 51–69. 10.1111/j.1744-6570.2001.tb00085.x

[B77] van der Vegt G. S. van de Vliert E. Huang X. (2005). Location-level links between diversity and innovative climate depend on national power distance. *Acad. Manage. J.* 48 1171–1182. 10.2307/20159736

[B78] van der Vegt G. S. van de Vliert E. Oosterhof A. (2003). Informational dissimilarity and organizational citizenship behavior: the role of intrateam interdependence and team identification. *Acad. Manage. J.* 46 715–727. 10.2307/30040663

[B79] Van Dyne L. Pierce J. L. (2004). Psychological ownership and feelings of possession: three field studies predicting employee attitudes and organizational citizenship behavior. *J. Organ. Behav.* 25 439–459. 10.1002/job.249

[B80] Vandewalle D. Van Dyne L. Kostova T. (1995). Psychological ownership: an empirical examination of its consequences. *Group Organ. Manage.* 20 210–226. 10.1177/1059601195202008

[B81] Wagner S. H. Parker C. P. Christiansen N. D. (2003). Employees that think and act like owners: effects of ownership beliefs and behaviors on organizational effectiveness. *Pers. Psychol.* 56 847–871. 10.1111/j.1744-6570.2003.tb00242.x

[B82] Wallace J. C. Butts M. M. Johnson P. D. Stevens F. G. Smith M. B. (2016). A multilevel model of employee innovation: understanding the effects of regulatory focus, thriving, and employee involvement climate. *J. Manag.* 42 982–1004. 10.1177/0149206313506462

[B83] Yoon S. K. Kim J. H. Park J. E. Kim C. J. Song J. H. (2020). Creativity and knowledge creation: the moderated mediating effect of perceived organizational support on psychological ownership. *Eur. J. Train. Dev.* 44 743–760. 10.1108/ejtd-10-2019-0182

[B84] Zhang Z. Min M. (2021). Organizational rewards and knowledge hiding: task attributes as contingencies. *Manag. Decis.* 59 2385–2404.

[B85] Zhou J. Yang J. Zhou X. (2021). Customer cooperation and employee innovation behavior: the roles of creative role identity and innovation climates. *Front. Psychol.* 12:639531. 10.3389/fpsyg.2021.639531 34149522 PMC8209253

[B86] Zweber Z. M. Henning R. A. Magley V. J. (2016). A practical scale for multi-faceted organizational health climate assessment. *J. Occup. Health Psychol.* 21 250–259. 10.1037/a0039895 26569133

